# Nationally representative surveys on cannabis use lack product details relevant to public health

**DOI:** 10.1016/j.dadr.2023.100134

**Published:** 2023-01-13

**Authors:** Sarah Stith, Jennifer Pearson, Franco Brockelman, Keenan Keeling, Branden Hall, Abigail S. Friedman

**Affiliations:** a Department of Economics, University of New Mexico, 1915 Roma Ave. NE, #1006B, Albuquerque, NM 87131, United States; b School of Public Health, University of Nevada, Rena, NV, United States; c MoreBetter, Ltd., Hyattsville, MD, United States; d Yale School of Public Health, Yale University, CT, United States

**Keywords:** Cannabis, Marijuana, Surveillance survey

## Abstract

**Objectives:**

Many annual, nationally representative US surveys that assess cannabis use do not collect information on product characteristics despite varying health risks and benefits. Capitalizing on a rich dataset of primarily medical users, the purpose of this study was to describe the degree of potential misclassification in clinically relevant cannabis use measures when primary mode of use is recorded but not product type.

**Methods:**

Analyses consider user-level data from the Releaf App™ database on product types, consumption modes, and potencies in a non-nationally representative sample of 26,322 cannabis administration sessions occurring in 2018, across 3,258 users. Proportions, means, and 95% confidence intervals were calculated and compared across products and modes.

**Results:**

Primary consumption modes were smoking (47.1%), vaping (36.5%), and eating/drinking (10.4%), with 22.7% of users reporting multiple modes of use. Moreover, mode of use did not signify a single product type: users reported vaping both flower (41.3%) and concentrates (68.7%). Of those who smoked cannabis, 8.1% reported smoking concentrates. Concentrates averaged 3.4 times higher tetrahydrocannabinol (THC) potency and 3.1 times higher cannabidiol (CBD) potency than flower.

**Conclusions:**

Cannabis consumers employ multiple consumption modes, and product type cannot be inferred from mode of use. With THC potencies markedly higher in concentrates, these findings underscore the importance of including information on cannabis product types and mode of use in surveillance surveys. Clinicians and policymakers need these data to inform treatment decisions and assess cannabis policies’ implications for population health.

## Highlights

• Nationally representative surveys measure cannabis use and not the scope of cannabis consumption.
• Cannabis consumers use a range of products with varying potencies and modes of consumption.
• These product characteristics are necessary for understanding effects of cannabis consumption.
• Understanding how cannabis is consumed would improve clinical use and regulatory policies.

## 1 Introduction

As more US states legalize medical and adult cannabis use, the range of cannabis products, consumption modes, and tetrahydrocannabinol (THC) and cannabidiol (CBD) potencies has expanded (Hasin et al., 2016; Kritikos and Pacula, 2022), introducing variation in cannabis use behaviors and related health implications. For example, self-reported therapeutic benefits and side effects vary with product, consumption mode, and potency (Stith et al., 2019). At the same time, while THC in flower is biologically limited, concentrated products can be almost 100% THC, potentially elevating risks for psychosis, neurotoxicity, and cardiotoxicity (Petrilli et al., 2022; Pierre et al., 2016; Rickner et al., 2017). Yet current, nationally representative US surveillance survey data on cannabis use typically omit clinically relevant details on mode of cannabis use, product type, and potency. Moreover, surveys covering some but not all of these details may introduce misclassification bias, because a single product type can correspond to multiple modes of use —e.g., smoking dried flower remains the most common route of administration/product type combination, but vaping dried flower or oils is increasingly prevalent (Wadsworth et al., 2022) and likely exposes users to less carbon monoxide and tar than smoking (Gieringer et al., 2004)—and vice versa. Given expanding product characteristics and potentially different mechanisms of action, understanding the level of detail required to avoid clinically relevant misclassification in cannabis survey data is critical to inform surveillance survey items, clinician practice, and regulation as states increasingly adopt new cannabis laws.

Annual, nationally representative US surveillance surveys that regularly assess cannabis use (Geissler et al., 2020) largely fail to differentiate product characteristics and consumption modes (e.g., the National Survey on Drug Use and Health (NSDUH) and National Health and Nutrition Examination Survey). The Behavioral Risk Factor Surveillance System (BRFSS) asks about primary consumption mode in its optional “Marijuana Module,” but does not capture multimodal use or product types, and many states omit that section. To date, few datasets contain the mix of variables necessary to understand the degree of misclassification that arises from assumptions about the association between mode of use and product type, even among medical cannabis users (Hammond et al., 2022).^1^ Despite this information's clinical relevance and importance for understanding cannabis policies’ health effects, calls for improved cannabis use measures often punt on this issue. Indeed, a recent expert consensus study proposing minimum standards for measuring cannabis use describes capturing “the wide range of cannabis products available” as an area for future work (Lorenzetti et al., 2022).To clarify the degree of clinically relevant misclassification that may occur when surveillance surveys omit cannabis product characteristics, we used self-reported data from Releaf App™, a large database of primarily medical cannabis administration sessions, to illustrate the range of products used, how product type and potency varied within modes of consumption, and the prevalence of multimodal use. These data offer a superior level of product and consumption mode detail relative to currently available, annual, nationally representative US surveillance surveys. Though Releaf data are neither designed to be nationally representative nor publicly available, their level of detail and orientation towards medical cannabis users has the potential to clarify clinically relevant information on mode of use and product type that may be consequential for population health. With medical use legal in about three quarters of the US, including details in nationally representative US surveillance surveys should be a priority, in order to evaluate current and future cannabis policies’ health effects.

## 2 Methods

*Data source & measures*. Data come from Releaf App™ (Releaf, 2022), a free app for tracking real-time, subjective positive and negative effects of cannabis use across products and consumption modes. The app was designed by coauthors Franco Brockelman, Keenan Keeling, and Branden Hall, and is owned by MoreBetter, Ltd., founded by the same subset of coauthors. Participants are not remunerated and the only reward for participation is greater insight into the therapeutic effectiveness of cannabis across product characteristics. The Releaf App requires users to enter their reason for use, cannabis product type (e.g., flower, concentrate, edible, tincture, topical, pill, or “other”), and, for users who specify flower or concentrate, their inhalation method (e.g., joint, pipe, or vape). Thus, the app restricts users to one type of product and consumption mode per cannabis administration session. Optional fields include product potency and the user's age, gender, and state.

We restricted our analyses to 2018 to capture broadly generalizable use patterns while avoiding potential changes in use triggered during the 2019 EVALI outbreak or the COVID-19 pandemic. Between 01/01/2018 and 12/31/2018 3621 users entered cannabis product and consumption information in 68,255 treatment events, where the number of treatment events in a single cannabis administration session reflects the number of symptoms being treated (e.g., a user specifying two symptoms during the same session has two treatment events). Collapsing treatments to the session-level (so that a session treating multiple symptoms at once—e.g., anxiety and muscle spasms—is presented as one observation rather than two medical treatment events), excluding people who self-reported their age as <18 or their location as outside the US, and dropping use sessions with incomplete data reduced our dataset to 3258 users who recorded 26,322 cannabis use sessions using 9693 unique products. The Online Supplemental Appendix contains additional details on sample and variable construction. Further information on the underlying session-level data is available by downloading the Releaf App or in prior publications using the Releaf App data (e.g., Stith et al., 2019).

*Analyses.* As users reported mode of use for only flower and concentrates, we created a mode variable assuming that edibles and tinctures were consumed orally; with topical and “other” products collapsed and coded as “other” modes. We created binary indicators for each product type, mode, and product-by-mode combination (i.e., capturing whether a session involved smoking flower, smoking concentrates, vaping flower, or vaping concentrates). Where potency was reported, separate THC and CBD percentage variables were generated for sessions involving flower and concentrates. (See the Online Supplemental Appendix for histograms of THC and CBD potencies for flower and concentrates, which were generated from the underlying session-level data). These data were then collapsed to the user level, summing the indicator variables, averaging user-level THC and CBD potency separately for flower and concentrates, and retaining users’ self-reported sex, age, and state variables, the last of which was used to generate Census Region indicators. Optional information not reported by users was coded as missing. Indicators capture whether a user ever reported each product and mode; a continuous variable gives the number of modes they reported; and categorical variables capture their primary mode of use as well as two product-by-mode variables – one for whether users reported only smoking flower, only smoking concentrates, or both, and the other for whether either of these product categories were ever reported vaped. We calculated means and 95% confidence intervals for each variable. All analyses were conducted using Stata version 15.1. The University of New Mexico Institutional Review Board deemed the project non-human-subjects research due to complete deidentification of the data.

## 3 Results

Overall, users reported an average of 28 and a median of 4 use sessions. The average user entered 11.1 distinct products, whereas the median user entered 4; 607 users entered only one session and one corresponding product. The majority of Releaf App users who reported demographic information were female (56.9%), ages 25–44, and living in the West (Table 1). Comparing outcome measures between those with missing versus non-missing demographic data suggests that those reporting demographics were more likely to vape, particularly concentrates (Online Supplemental Appendix Table 1).

**Table 1:** Sample description and results.

Variable	Obs	Proportion	95% CI Lower Bound	95% CI Upper Bound
Male {0,1}:	1920	43.1%	40.9%	45.3%
Age Category:				
Ages 18–24	1841	19.1%	17.4%	21.0%
Ages 25–34	1841	31.6%	29.5%	33.7%
Ages 35–44	1841	27.2%	25.2%	29.3%
Ages 45–54	1841	13.0%	11.6%	14.7%
Ages 55–64	1841	7.2%	6.1%	8.5%
Ages 65-plus	1841	1.8%	1.3%	2.6%
Census Region:				
West	1593	38.4%	36.0%	40.8%
Northeast	1593	26.6%	24.4%	28.8%
Southeast	1593	26.4%	24.3%	28.6%
Midwest	1593	8.7%	7.4%	10.1%
Primary Mode of Use:				
Vaping	3258	36.5%	34.9%	38.2%
Smoking	3258	47.1%	45.3%	48.8%
Eating/Drinking	3258	10.4%	9.4%	11.5%
Other	3258	1.4%	1.1%	1.9%
Number of Modes:				
One	3258	77.3%	75.9%	78.8%
Two	3258	17.1%	15.8%	18.4%
Three-plus	3258	5.6%	4.8%	6.4%
Any Use:				
Any Vaping	3258	47.3%	45.6%	49.0%
Any Smoking	3258	56.3%	54.6%	58.0%
Any Edible	3258	12.0%	10.9%	13.2%
Any Tincture	3258	10.5%	9.5%	11.6%
Any Other	3258	3.7%	3.1%	4.5%
Product Vaped:				
Vaped Flower & Concentrates	1542	10.2%	8.8%	11.9%
Vaped Flower Only	1542	31.3%	29.0%	33.6%
Vaped Concentrates Only	1542	58.5%	56.0%	60.9%
Product Smoked:				
Smoked Flower & Concentrates	1833	3.5%	2.8%	4.5%
Smoked Flower Only	1833	91.8%	90.5%	93.0%
Smoked Concentrates Only	1833	4.6%	3.8%	5.7%
THC Potency†:				
Flower	956	17.9%	17.4%	18.4%
Concentrates	652	60.5%	58.5%	62.5%
CBD Potency†:				
Flower	680	7.0%	6.5%	7.5%
Concentrates	456	21.4%	19.3%	23.4%

Notes: Data include users aged 18 and older, who recorded at least one cannabis use session in the Releaf App between 01/01/2018 and 12/31/2018. "Other" *Primary Mode of Use* and *Any Use* includes topicals and pills. All variables are categorical and have multiple mutually exclusive outcomes except for the *Any Use* variables and *Male*, which are dichotomous {0,1}. Agresti-Coull 95% confidence intervals are reported (Agresti and Coull, 1998). Due to reporting differences, the observation counts differ within the table above. Product type is required reporting and reflects the complete sample of users who completed sessions in 2018. Inhalation method is only required reporting for individuals who enter flower or concentrates as the product type. Product Vaped is restricted to those users who reported vaping a flower and/or concentrates and Product Smoked is restricted to those users who reported smoking flower and/or concentrates. Demographic information and THC and CBD potency are not required reporting as reflected by the smaller counts of users reporting this information.

† Potency reporting is optional, and only consistently reported as a percentage for THC and CBD. Although milligrams are commonly used for other consumption methods like edibles, the app does not track total product weight, so we can only generate comparable potency measures for flower and concentrates.

Smoking was the most common primary consumption mode, but primary mode of use concealed substantial multimodal cannabis consumption: 17.1% (95% CI: 15.8%, 18.4%) of users reported using two modes, while 5.6% (95% CI: 4.8%, 6.4%) reported three or more modes over the 12-month period. As far as “any reported use,” smoking cannabis was the most common (56.3%; 95% CI: 54.6%, 58.0%), followed by vaping (47.3%; 95% CI: 45.6%, 49.0%), consuming edibles (12.0%; 95% CI:10.9%, 13.2%), ingesting tinctures (10.5%; 95% CI: 9.5%, 11.6%), or using topicals or pills (3.7%; 95% CI: 3.1%, 4.5%).

Product type varied within consumption mode. Although concentrates were the most common product vaped, almost a third of users reported vaping only flower (31.3%; 95% CI: 29.0%, 33.6%), with another 10.2% (95% CI: 8.8%, 11.9%) reporting vaping flower and concentrates. While smoking overwhelmingly involved flower, 4.6% (95% CI: 3.8%, 5.7%) of self-reported cannabis smokers reported smoking only concentrates, and 3.5% (95% CI: 2.8%, 4.5%) reported that they smoked flower in some sessions and concentrates in others. Thus, inferring product type from primary mode of use alone here would introduce misclassification bias.

THC percentages were 3.4 times higher for concentrates (60.5%; 95% CI: 58.5%,62.5%) than for flower (17.9%; 95% CI: 17.4%, 18.4%) and CBD percentages were 3.1 times higher for concentrates (21.4%; 95% CI: 19.3%, 23.4%) than for flower (7.0%; 95% CI: 6.5%,7.5%).

## 4 Discussion

With a fifth of users reporting multiple modes of use over a 12-month period and almost a third of cannabis vapers reporting that they vape flower but not concentrates, “primary mode of use” variables conceal substantial, clinically important heterogeneity in mode of use and product type. These findings suggest that asking consumers about any recent cannabis use without details on product type, as is common in annual US surveillance surveys, or relying on “primary consumption mode” as in the BRFSS Marijuana Module, could mask crucial differences in the health risks and clinical benefits of cannabis use, especially among frequent cannabis users.

Results also suggest that users may be able to accurately report THC and CBD concentration: self-reported potencies in the Releaf App data are consistent with Washington's state dispensary sales data, which found an almost identical THC potency ratio between concentrates and flower (3.3) and similar average potencies by product type: 20.6% for flower, 68.7% for concentrates. CBD potencies were higher than in the Releaf data at 7.0% for flower and 21.4% for concentrates, versus 0.34% and 1.8%, respectively in Washington state (Smart et al., 2017). Slightly lower THC values in our sample combined with much higher CBD levels may reflect Releaf App users skewing towards medical rather than recreational use or differences between Washington state and the wider US.

This study's primary limitations relate to sample selection. First, Releaf App data are not designed to be nationally representative and may overrepresent individuals with legal access to cannabis (if legal risks deter participation) and medical users, as the application was created to monitor the therapeutic effects of cannabis use. Both the 2018 Releaf App data and 2018 NSDUH for past year cannabis users ages 18 and older show greater use among 18–35 year-olds than older age-groups (50.7% and 54.9% for NSDUH and Releaf, respectively) but with Releaf's sample more female (56.9%) than NSDUH's (43.2%), consistent with more medical use. Reassuringly, this reinforces our conclusion: multimodal use and variation in product choice within mode of use among medical cannabis users underscores the need for surveillance data on cannabis product characteristics to inform clinician decision-making. Second, users may not record all their cannabis use sessions, which could underestimate the prevalence of multimodal use and breadth of products and modes. Reassuringly, such bias would strengthen our conclusion that omitting these data from surveillance surveys masks vital information on the health consequences of cannabis use, policies, and commercialization. Thirdly, as potency testing is not required for home-cultivated or illicit cannabis products, THC and CBD values likely came from labels on dispensary-sourced products. Lastly, the data collection method only includes individuals capable of and interested in tracking their health information using an mobile app. This mode of data collection may skew our sample towards younger, more educated, and wealthier individuals (Carroll et al., 2017; Krebs and Duncan, 2015). Despite this caveat, app-based data collection also offers an important advantage: by collecting product characteristics and mode of use concurrent with use, in real time, Releaf users’ reporting is less subject to recall bias that typical surveys, and thus more likely to capture details that may be important clinically and for public health.

## 5 Conclusion

Our results suggest that key US surveillance surveys’ cannabis use questions fail to capture vital information needed to assess the public health effects and clinical implications of cannabis use and related policies. As the cannabis product landscape is changing rapidly, nationally representative data on product types, consumption modes, and potencies are needed to understand the effects of legalization and commercialization on cannabis product characteristics and population health. Evidence that adults consume different product types via a single mode of use and that potencies vary markedly by cannabis product type highlights a need to track product types and modes of use in population surveillance surveys, in order to address concerns about negative sequelae from higher potency products and combustible use, questions about medicinal regimens and their effectiveness, and risks from adulterated cannabis concentrates (as implicated in the US's 2019 outbreak of vaping-associated lung injuries (Friedman and Morean, 2021).) Such information will inform clinician decision making, cannabis policies, product safety standards, and targets for policy interventions to reduce high risk cannabis use and protect public health.

## Role of funding source

Nothing declared.

## CRediT authorship contribution statement

**Sarah Stith:** Visualization, Formal analysis, Writing – review & editing. **Jennifer Pearson:** Visualization, Writing – review & editing. **Franco Brockelman:** Software, Resources, Writing – review & editing. **Keenan Keeling:** Software, Resources, Writing – review & editing. **Branden Hall:** Software, Resources, Writing – review & editing. **Abigail S. Friedman:** Visualization, Writing – review & editing.

## Declaratlion of Competing Interest

No conflicts declared for authors Abigail Friedman, Jennifer Pearson, and Sarah Stith. Authors Franco Brockelman, Keenan Keeling, and Branden Hall designed and own the Releaf App™ and provided the data for this project without remuneration.

## Appendix Supplementary materials

Image, application 1

## Footnotes

1 Detailed retrospective data on cannabis use are available through the International Cannabis Policy Study (ICPS), which collects vital cannabis use data but relies on non-probability samples with far fewer respondents than the BRFSS or NSDUH. ICPS investigators have affirmed a need for US “benchmark data” to compare to ICPS results (Hammond et al., 2022).
