# Supplementary material for: Nationally representative surveys on cannabis use lack product details relevant to public health
Source: Drug Alcohol Depend Rep. 2023 Jan 13;6:100134. doi: 10.1016/j.dadr.2023.100134 (PMC10040319; doi:10.1016/j.dadr.2023.100134)
Supplement: Supplementary file 1 [file mmc1.docx]

Online Supplemental Appendix to “Nationally Representative Surveys on Cannabis Use Lack Product Details Relevant to Public Health”

DATA APPENDIX

Data, Sample, and Variable Construction

I. Self-reported information on product, consumption mode, and demographics was collected in date-stamped cannabis administration sessions prior to beginning cannabis consumption. The diagram below shows how a Releaf App user proceeds through the app from initially opening the app through entering product and consumption mode information and beginning a cannabis consumption session. Note that users can track the effect of a single cannabis product (and consumption mode) on multiple symptoms during the same cannabis administration session. Each time a user attempts to treat a specific symptom is counted as a treatment event, i.e., an observation. (Only information used in this study is included in the diagram; endnotes add additional details.)

Optional Fields:

- THC potency^c^
- THC % or mg^d^
- CBD potency^c^
- CBD % or mg^d^
- State or Country^e^
- Age
- Gender^f^

After recording symptom and starting symptom intensity, app users begin cannabis consumption…^g^

Inhalation Method^b^ (required for flower and concentrates):

- Joint
- Pipe
- Vape

Cannabis Product

(required):

- Flower
- Concentrates
- Edibles
- Tinctures
- Topicals
- Pills^a^

The initial sample session dates ranged from 06/06/2016 to 01/17/2022 and included 312,903 treatment events reported by 15,916 app users.

II. We dropped observations with year ≠ 2018, age<18, country ≠ U.S., leaving us with a sample of 62,236 symptom-level treatment events reported by 3,369 users. We dropped an additional 2,219 treatment events and 111 users who did not enter complete product information, i.e., flower and concentrates users who did not designate required inhalation mode information. (This also means the session itself was never initiated, as inhalation method is required information to proceed with the cannabis administration session.) The final sample of treatment events included 60,017 such events recorded by 3,258 users.

III. As only one product and inhalation method can be indicated per session but a single session can treat multiple symptoms, we keep only one observation per cannabis administration session. This left us with 26,322 sessions (rather than treatment events) in which 9,693 cannabis products were reported as consumed by the 3,258 users in our sample. We used these data to create 13 dichotomous {No = 0, Yes = 1} product, mode, and product-by-mode variables, and potency variables for individuals who specified the potency as a percentage of total weight, based on the following underlying questions:

A. Was the product consumed …? {0,1}

1. Flower
2. Concentrates
3. Edibles
4. Tinctures
5. Other (Topicals, Pills)

B. Was the consumption mode …? {0,1}

1. Smoking (for flower or concentrates, consumption mode =joint or pipe)
2. Vaping (for flower or concentrates, consumption mode = joint or pipe)
3. Eating/Drinking (follows directly from product type for edibles or tinctures)
4. Other (follows directly from product type for topicals or pills)

C. What was smoked? {0,1}

1. Product = flower; consumption mode = smoking
2. Product = concentrates, consumption mode = smoking

D. What was vaped? {0,1}

1. Product = flower, consumption mode = vaping
2. Product = concentrates, consumption mode = vaping

E. In what units are THC and CBD potency reported?

1. Percent
2. Milligrams

F. How much THC was reported?

G. How much CBD was reported?

IV. We collapsed the data by user, summing the number of times each product, mode or product-by-mode was reported by a given person, calculating the user-level mean reported THC and CBD potencies for flower and for concentrates for observations with potencies recorded as a percent, and keeping the session-invariant state, age, and gender variables (already user-level).

V. At the user level and in line with the questions listed below, we created 9 dichotomous {No = 0, Yes = 1} variables and 4 categorical variables from the existing product, mode, and product-by-mode variables; generated mean THC and CBD separately for flower and and concentrates; and identified age groups, gender, and Census Region from self-reported age, gender, and state names.

A. Was … consumed at least once? {0,1}

1. Flower
2. Concentrates
3. Edibles
4. Tinctures
5. Other (Topicals, Pills)

B. Was … used as a consumption mode at least once? {0,1}

1. Smoking
2. Vaping
3. Eating/Drinking
4. Other

C. Categorical Variables

1. How many different consumption modes did user report? (categorical: 1,2,3+)
2. Was … the most frequent consumption mode? (categorical)

- Smoking
- Vaping
- Eating/Drinking
- Other

1. What products were smoked? (categorical)

- Flower & Concentrates
- Flower Only
- Concentrates Only

1. What products were vaped? (categorical)

- Flower & Concentrates
- Flower Only
- Concentrates Only

VI. We report sample proportions and means, shown in Table 1, and calculate 95% confidence intervals in line with Agresti-Coull (Agresti and Coull, 1998), as recommended by Brown, Cai, and Dasgupta (2001) for binomially distributed data and samples of more than 40 observations.

References:

​​​​​​Agresti​, ​​A.​, ​​​Coull​, ​​B.A.​, ​​1998​. ​​Approximate is better than “exact” for interval estimation of binomial proportions​. ​​Am. Stat.​ ​​52​, ​​119​–​​126​. doi:​​10.1080/00031305.1998.10480550​.

Brown​, ​​L.D.​, ​​​Cai​, ​​T.T.​, ​​​DasGupta​, ​​A.​, ​​2001​. ​​Interval estimation for a binomial proportion​. ​​Stat. Sci.​ ​​16​, ​​101​–​​133​. doi:​​10.1214/ss/1009213286​.

Endnotes:

^a^ Pills were first added in July 2018 due to user demand for this option.

^b^ Inhalation method does not include “dabbing” as the app is focused on medical use.

^c^ As THC and CBD potency testing is expensive and required only for legal, commercial cannabis products, our THC and CBD measures likely do not capture the THC and CBD potencies of illicit or home-cultivated products. We also exclude THC and CBD levels above 35% for flower from the underlying session-level data, as these are biologically impossible. Histograms for the cleaned, session-level THC and CBD data are available in the Online Supplemental Appendix Figure 1 below.

^d^ THC and CBD units can be reported as a percent or in milligrams. Percent is much more widely reported, allows for comparability across products, and is the manner in which THC and CBD are reported for flower and concentrates, with the latter the product of most concern in terms of historically unprecedented THC levels. Because THC and CBD are primarily reported in milligrams for edibles and tinctures, without a total weight specified, we did not study potency for those products.

^e^ While only 48 states and the District of Columbia are reported in our data, state is only reported by 49% of our sample. Thus, it is possible that our sample includes data from all 50 states. Similarly, as we omit only users who report being outside the United States, the analytic sample could include some individuals from outside the U.S. if they did not specify their location.

^f^ The variable is called “gender” in the app, but the options available are “female,” “male,” or “write-in.”

^g^ Users touch a button to signal beginning a cannabis administration session. During the session (of unspecified length) they can record their symptom as many times as they desire and choose among 47 side effects they may be experiencing. To end a session, they enter an ending symptom intensity level. Symptom intensity is measured on a 0-10 analog scale, with 10 being very intense and 0 being no discernable symptom level. Fifty-three symptoms are available for selection and users can track multiple symptoms in a single session. The app provides feedback across products and overtime on symptom relief, side effects, time-to-relief, and dosing.

TABLE & FIGURE

Table 1: Tests for Differences among Users Reporting versus Not Reporting Demographic Information

|  | Demographic Information Reported | | Demographic Information Not Reported | |  |
| --- | --- | --- | --- | --- | --- |
| Variable | Obs | Proportion | Obs | Proportion | P-Value for Difference |
| Primary Mode of Use: |  |  |  |  | 0.036 |
| Vaping | 1,457 | 0.39 | 1,264 | 0.35 |  |
| Smoking | 1,457 | 0.45 | 1,264 | 0.48 |  |
| Eating/Drinking | 1,457 | 0.10 | 1,264 | 0.12 |  |
| Other | 1,457 | 0.02 | 1,264 | 0.01 |  |
| Number of Modes: |  |  |  |  | 0.107 |
| One | 1,457 | 0.76 | 1,264 | 0.81 |  |
| Two | 1,457 | 0.18 | 1,264 | 0.15 |  |
| Three-plus | 1,457 | 0.06 | 1,264 | 0.04 |  |
| Any Use: |  |  |  |  |  |
| Any Vaping | 1,457 | 0.50 | 1,264 | 0.44 | 0.002 |
| Any Smoking | 1,457 | 0.55 | 1,264 | 0.56 | 0.106 |
| Any Edible | 1,457 | 0.12 | 1,264 | 0.13 | 0.757 |
| Any Tincture | 1,457 | 0.11 | 1,264 | 0.10 | 0.486 |
| Any Other | 1,457 | 0.05 | 1,264 | 0.02 | 0.021 |
| Product Vaped: |  |  |  |  | <0.001 |
| Vaped Flower & Concentrates | 733 | 0.11 | 553 | 0.08 |  |
| Vaped Flower Only | 733 | 0.21 | 553 | 0.44 |  |
| Vaped Concentrates Only | 733 | 0.68 | 553 | 0.47 |  |
| Product Smoked: |  |  |  |  | 0.039 |
| Smoked Flower & Concentrates | 797 | 0.05 | 703 | 0.02 |  |
| Smoked Flower Only | 797 | 0.91 | 703 | 0.92 |  |
| Smoked Concentrates Only | 797 | 0.04 | 703 | 0.06 |  |
| THC Potency: |  |  |  |  |  |
| Flower | 457 | 18.38 | 319 | 17.34 | 0.080 |
| Concentrates | 364 | 59.93 | 176 | 61.18 | 0.546 |
| CBD Potency: |  |  |  |  |  |
| Flower | 323 | 3.34 | 221 | 5.23 | 0.064 |
| Concentrates | 249 | 13.57 | 128 | 11.85 | 0.650 |

Notes: Notes: Data include users aged 18 and older who recorded at least one session in the Releaf App between 01/01/2018 and 12/31/2018. Other Product Type includes Edibles, Tinctures, Topicals and Pills. Agresti-Coull (1998) 95% confidence intervals are reported.^11^ We use chi-squared tests to test for differences in categorical outcomes across the two groups with p-values reported in the last column. All variables have multiple mutually exclusive outcomes except for the "Any Use" variables, which are dichotomous {0,1}, and the potency variables which are in percentages [0,100].

Figure 1: Histograms of THC and CBD Percentages for Flower and Concentrates

Notes: The histograms show the reporting frequency for different THC and CBD potencies (percent) for Flower and Concentrates. THC and CBD in flower were capped at 35% due to the biological limitations of the plant. THC and CBD levels were allowed to approach 100 percent for concentrates, along with product labeling in commercial markets.
